# Genetically supported causality between gut microbiota, immune cells, and ischemic stroke: a two-sample Mendelian randomization study

**DOI:** 10.3389/fmicb.2024.1402718

**Published:** 2024-06-04

**Authors:** Han Shuai, Zi Wang, Yinggang Xiao, Yali Ge, Hua Mao, Ju Gao

**Affiliations:** ^1^Northern Jiangsu People’s Hospital Affiliated to Yangzhou University, Yangzhou, China; ^2^Peking University People’s Hospital, Qingdao Women and Children’s Hospital, Qingdao University, Qingdao, China

**Keywords:** Mendelian, gut microbiota, immunity, ischemic stroke, causality

## Abstract

**Background:**

Previous studies have highlighted a robust correlation between gut microbiota/immune cells and ischemic stroke (IS). However, the precise nature of their causal relationship remains uncertain. To address this gap, our study aims to meticulously investigate the causal association between gut microbiota/immune cells and the likelihood of developing IS, employing a two-sample Mendelian randomization (MR) analysis.

**Methods:**

Our comprehensive analysis utilized summary statistics from genome-wide association studies (GWAS) on gut microbiota, immune cells, and IS. The primary MR method employed was the inverse variance-weighted (IVW) approach. To address potential pleiotropy and identify outlier genetic variants, we incorporated the Mendelian randomization pleiotropy residual sum and outlier (MR-PRESSO) technique, along with MR-Egger regression. Heterogeneity was assessed using Cochran’s Q-test. Additionally, leave-one-out analysis was conducted to pinpoint any individual genetic variant influencing the observed causal associations. Finally, a reverse MR analysis was performed to explore the potential of reverse causation.

**Results:**

Our investigation revealed four gut microbial taxa and 16 immune cells with a significant causal relationship with IS (*p* < 0.05). Notably, two bacterial features and five immunophenotypes were strongly associated with a lower IS risk: genus.*Barnesiella.id.944* (OR: 0.907, 95% CI: 0.836–0.983, *p* = 0.018), genus.*LachnospiraceaeNK4A136group.id.11319* (OR: 0.918, 95% CI: 0.853–0.983, *p* = 0.988), Activated & resting Treg % CD4++ (OR: 0.977, 95% CI: 0.956–0.998, *p* = 0.028). Additionally, significant associations between IS risk and two bacterial features along with eleven immunophenotypes were observed: genus.*Paraprevotella.id.962* (OR: 1.106, 95% CI: 1.043–1.172, *p* < 0.001), genus.*Streptococcus.id.1853* (OR: 1.119, 95% CI: 1.034–1.210, *p* = 0.005), CD127 on granulocyte (OR: 1.039, 95% CI: 1.009–1.070, *p* = 0.011). Our analyses did not reveal heterogeneity based on the Cochrane’s Q-test (*p* > 0.05) nor indicate instances of horizontal pleiotropy according to MR-Egger and MR-PRESSO analyses (*p* > 0.05). Furthermore, the robustness of our MR results was confirmed through leave-one-out analysis.

**Conclusion:**

Our study provides further evidence supporting the potential association between gut microbiota and immune cells in relation to IS, shedding light on the underlying mechanisms that may contribute to this condition. These findings lay a solid foundation for future investigations into targeted prevention strategies.

## 1 Introduction

Ischemic stroke (IS), a prevalent neurological disorder, results from inadequate cerebral blood flow, leading to neuronal tissue hypoxia and ischemia (Zhu et al., 2022). Globally, approximately 80% of all stroke cases are attributed to ischemic stroke, presenting a significant public health challenge (Herpich and Rincon, 2020; Rabinstein, 2020). The substantial incidence rate and profound implications on disability and mortality underscore its importance. Clinically, ischemic stroke manifests as an acute impairment of brain function, with progression and prognosis contingent upon the affected region and extent of cerebral damage (Feske, 2021). Given the unsatisfactory therapeutic results and substantial burden linked to IS, it is crucial to ascertain potential underlying factors that contribute to its onset.

In the exploration of risk factors for ischemic stroke, researchers have increasingly emphasized the significance of gut microbiota (Pluta et al., 2021; Peh et al., 2022) and immune cells (Qiu et al., 2021). Gut microbiota, a vast microbial community within the human body, is closely linked to host health, influencing the integrity and function of the blood-brain barrier (BBB). Metabolites like short-chain fatty acids (SCFAs) regulate BBB permeability and stability, and the activation of the autonomic nervous system, such as the vagus nerve, by gut microbiota also affects BBB function (Long et al., 2022; Zhang et al., 2023). Disruption of the BBB is a crucial pathological feature of ischemic stroke, leading to complications such as edema, hemorrhage, and inflammation, exacerbating brain damage (Battaglini et al., 2020).

Immune cells, vital for the body’s defense system, play a crucial role in ischemic stroke through interactions with brain endothelial cells and the release of inflammatory factors. Factors like LAMs, IL-1β, and TNF-α influence the permeability and integrity of cerebral blood vessels, while surface molecules or released inflammatory factors like CD40, CD40L, IL-6, and TNF-α regulate vascular tone in brain blood vessels by influencing the synthesis and release of nitric oxide (NO) (Iadecola et al., 2020; Xu et al., 2020; Endres et al., 2022). Both gut microbiota and immune cells have been identified as potential contributors to ischemic stroke development, influencing disease occurrence through interactions with the nervous system (Nam, 2019; Hu et al., 2022; Huang et al., 2022; Wang et al., 2022).

Mendelian randomization (MR), an effective approach to infer causality, employs genetic variants as instrumental variables (IVs) to investigate the causal impact of exposure on outcomes (Bowden and Holmes, 2019; Birney, 2022). In this study, gut microbiota and immune cells were selected as exposure variables, with IS as the outcome variable for MR analysis. The goal was to explore potential causal relationships, providing a theoretical foundation for further investigations into the intricate mechanisms and risk factors associated with IS.

## 2 Materials and methods

### 2.1 Ethics approval statement

The summary-level data utilized in this research is available for download. The genome-wide association studies (GWAS) conducted for this study received approval from the relevant institutions, adhering to ethical guidelines.

### 2.2 Study design

**FIGURE 1:**
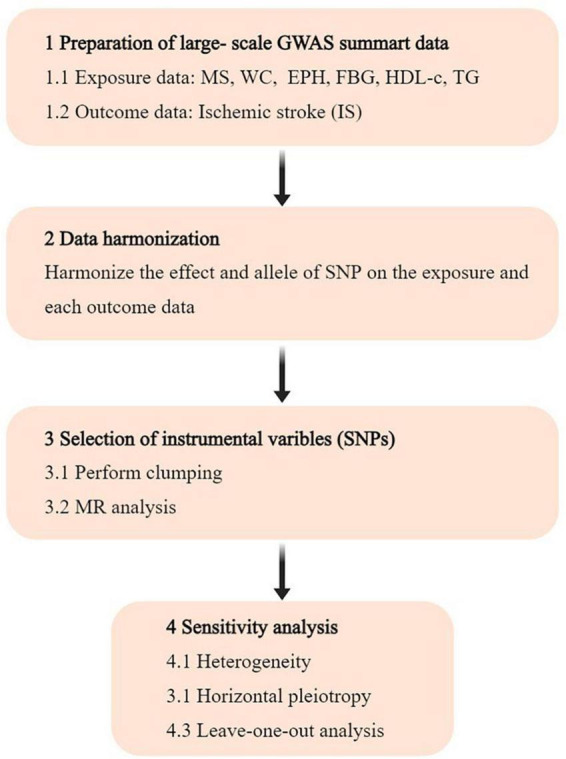
Flowchart of the study. GWAS, genome-wide association study; SNP, single nucleotide polymorphism; MR, Mendelian randomization.

The investigation focused on gut microbiota and immune cells as exposures, with ischemic stroke (IS) as the outcome. All data analyzed were sourced from publicly accessible GWAS. Single nucleotide polymorphisms (SNPs) linked to gut microbial taxa and immune cells were extracted and utilized as IVs. A two-sample MR analysis was conducted using summary-level data from GWAS of gut microbiota, immune cells, and IS. The study’s flow chart is illustrated in Figure 1.

### 2.3 Exposure data of gut microbiota and immune cells

Relevant data on gut microbiota were extracted from the MiBioGen consortium’s comprehensive GWAS study, involving 18,340 individuals across 24 cohorts. Utilizing 16S rRNA gene sequencing profiles (Kurilshikov et al., 2021), the analysis focused on 211 taxa, including 131 genera, 35 families, 20 orders, 16 classes, and 9 phyla for mapping microbiome quantitative trait loci. Information on immune traits’ summary statistics was publicly accessible through the GWAS Catalog, with accession numbers ranging from GCST0001391 to GCST0002121 (Orrù et al., 2020). A total of 731 immunophenotypes were analyzed, covering parameters such as absolute cell (AC) counts (*n* = 118), median fluorescence intensities (MFI) indicating surface antigen levels (*n* = 389), morphological parameters (MP) (*n* = 32), and relative cell counts (RC) (*n* = 192). MFI, AC, and RC features encompassed B cells, CDCs, mature T cell stages, monocytes, myeloid cells, T-cell, B-cell, and Natural Killer cell (TBNK) panels, and Treg panels. Additionally, the MP feature included cytotoxic T cells (CDC) and TBNK panels. The initial GWAS analysis on immune traits involved 3,757 individuals of European descent, with no overlap in cohorts. Approximately 22 million SNPs were imputed using a Sardinian sequence-based reference panel to enhance genotyping data (Sidore et al., 2015). Associations were examined while accounting for potential confounding factors such as sex, age, and age squared.

### 2.4 Outcome data of ischemic stroke

Genome-wide association studies summary statistics for genetic associations related to IS were obtained from the largest GWAS meta-analysis conducted by the Malik R Lab (Malik et al., 2018). This extensive study included 440,328 individuals of European descent, comprising 34,217 cases and 406,111 controls. Analysis was performed on approximately 7.5 million variants following quality control measures and imputation.

### 2.5 Genetic instruments selection and harmonization

To ensure the reliability and precision of outcomes, a thorough quality check was conducted on SNPs to derive compliant IVs. The selection criteria for SNPs were as follows: (A) a strong association with exposures; (B) no correlation with confounding factors; (C) association with outcomes influenced by the exposures (Ference et al., 2021). Given the limited number of eligible IVs (genome-wide statistical significance threshold, *p* < 5 × 10^–8^), a locus-wide significance threshold (*p* < 5 × 10^–5^) was adopted for a more comprehensive outcome (Georgakis and Gill, 2021). To address linkage disequilibrium (LD), a clumping method was employed with parameters set at *r*^2^ = 0.001 and kb = 10,000. Subsequently, the robustness of the chosen SNPs was assessed by computing the F statistics, utilizing the formula: *F = R*^2^*(N-k-1)/[(1-R*^2^*)k]*. In this equation, *R*^2^ represents the proportion of variability explained by each SNP, N denotes the size of our GWAS sample, and k indicates the number of SNPs. A value of 10 for the F statistic suggests a lack of substantial evidence for instrument bias (Burgess et al., 2011). This comprehensive approach aimed to enhance the selection and harmonization of genetic instruments, ensuring the credibility of the subsequent analyses.

### 2.6 Statistical analysis

The primary MR analysis utilized the inverse variance-weighted (IVW) method. To assess the reliability of significant findings, sensitivity analyses were conducted using MR-Egger, weighted median, weighted mode, and simple mode (Vaucher et al., 2018). Cochran’s Q statistic and corresponding *p*-values were employed to assess heterogeneity among selected IVs. In cases where the null hypothesis was not supported, random effects IVW was used instead of fixed-effects IVW. To address potential horizontal pleiotropy, the MR-Egger approach was applied, indicating horizontal multiplicity if the intercept term was statistically significant. The MR-PRESSO method from the MR-PRESSO package (Zheng et al., 2022), a robust technique, was utilized to identify and exclude potential outliers with horizontal pleiotropic effects that could significantly influence estimation results. Scatter plots and funnel plots were employed to assess data integrity, with the former indicating that outliers did not substantially impact results. Meanwhile, funnel plots demonstrated no heterogeneity, confirming the robustness of the correlation. Additionally, a reverse causality analysis was conducted to evaluate potential reverse causal relationships. All analyses were performed using R version 4.3.1, incorporating packages such as “TwoSampleMR,” “MRPRESSO,” and “MendelianRandomization.” The corresponding codes for these analyses can be found in Supplementary Table 1.

## 3 Results

### 3.1 Selection of instrumental variables

In the initial phase, a comprehensive total of 13,749 single nucleotide polymorphisms (SNPs) for gut microbiota and 18,620 SNPs for immune cells were identified as potential instrumental variables (IVs) through large-scale GWAS, employing a locus-wide significance level of *p* < 1 × 10^–5^. These SNPs were carefully selected, excluding palindromic variants, as detailed in Supplementary Tables 2, 3. Following clumping and harmonization procedures, a refined set of 1,515 SNPs (*p* < 1 × 10^–5^) for gut microbiota and 18,620 SNPs (*p* < 1 × 10^–5^) for immune cells emerged as instrumental variables. The F-statistics for these IVs consistently surpassed the threshold of 10, indicating the absence of weak instrument bias. Crucial information regarding the SNPs’ key characteristics, such as effect allele, alternate allele, beta value, standard error, and *p*-value, was systematically collected for further analysis (Supplementary Tables 4, 5).

### 3.2 Causal effects of gut microbiota on ischemic stroke

**FIGURE 2:**
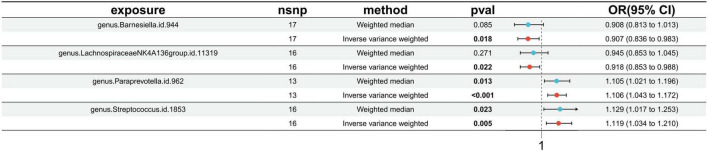
Forest plots presenting the Mendelian randomization findings of gut microbiota taxa associated with ischemic stroke causation. OR, odds ratio; CI, confidence interval; IVW, inverse variance weighted.

Utilizing the IVW method, our analysis identified four causal associations between gut microbiota features and traits related to IS (Supplementary Table 6). The IVW results indicated that an increased genetic predisposition to the genus. *Paraprevotella.id.962* (OR: 1.106, 95% CI: 1.043–1.172, *p* < 0.001), and genus. *Streptococcus.id.1853* (OR: 1.119, 95% CI: 1.034–1.210, *p* = 0.005) were associated with an elevated risk of IS. Conversely, a lower risk of IS was observed in relation to the genetically predicted abundance of genus.*Barnesiella.id.944* (OR: 0.907, 95% CI: 0.836–0.983, *p* = 0.018), and genus.*LachnospiraceaeNK4A136group.id.11319* (OR: 0.918, 95% CI: 0.853–0.983, *p* = 0.988) (Figure 2).

### 3.3 Causal effects of immune cells on ischemic stroke

**FIGURE 3:**
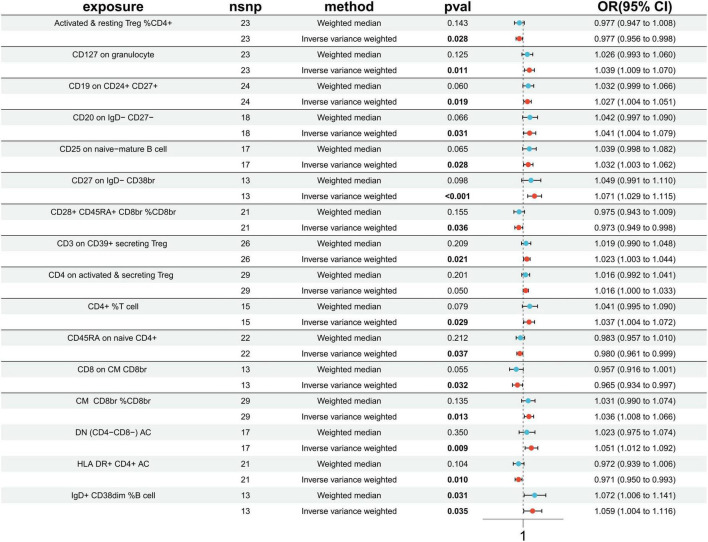
Forest plots presenting the Mendelian randomization findings on immune cells associated with ischemic stroke causality. OR, odds ratio; CI, confidence interval; IVW, inverse variance weighted.

Utilizing the IVW method, our analysis uncovered a total of 16 causal associations between immune cell features and traits related to IS. These associations comprised 5 in the B cell panel, 2 in the TBNK panel, 5 in the Treg panel, and 4 in the maturation stages of the T cell panel (Supplementary Table 7). The IVW analysis results indicated that an increased genetic abundance of CD127 on granulocyte (OR: 1.039, 95% CI: 1.009–1.070, *p* = 0.011), CD19 on CD24+ CD27+ (OR: 1.027, 95% CI: 1.004–1.051, *p* = 0.019), CD20 on IgD- CD27- (OR: 1.041, 95% CI: 1.004–1.079, *p* = 0.031), CD25 on naive-mature B cell (OR: 1.032, 95% CI: 1.003–1.052, *p* = 0.028), CD27 on IgD- CD38br (OR: 1.071, 95% CI: 1.029–1.115, *p* < 0.001), CD3 on CD39+ secreting Treg (OR: 1.023, 95% CI: 1.003–1.044, *p* = 0.021), CD4 on activated & secreting Treg (OR: 1.016, 95% CI: 1.000–1.033, *p* = 0.050), TD CD4+ %T cell (OR: 1.037, 95% CI: 1.004–1.072, *p* = 0.029), CM CD8br % CD8br (OR: 1.036, 95% CI: 1.008–1.066, *p* = 0.013), DN (CD4-CD8-) AC (OR: 1.051, 95% CI: 1.012–1.092, *p* = 0.009), IgD+ CD38dim %B cell (OR: 1.059, 95% CI: 1.004–1.116, *p* = 0.035) were associated with a higher risk of IS. Conversely, the genetically predicted abundance of Activated & resting Treg %CD4+ (OR: 0.977, 95% CI: 0.956–0.998, *p* = 0.028), CD28+ CD45RA+ CD8br %CD8br (OR: 0.973, 95% CI: 0.949–0.998, *p* = 0.036), CD45RA on naive CD4+ (OR: 0.980, 95% CI: 0.961–0.999, *p* = 0.037), CD8 on CM CD8br (OR: 0.965, 95% CI: 0.934–0.997, *p* = 0.032), HLA DR+ CD4+ AC (OR: 0.971, 95% CI: 0.950–0.993, *p* = 0.010) were correlated with a reduced risk of IS (Figure 3).

### 3.4 Pleiotropy, heterogeneity, sensitivity, and reverse analysis

The results of both the IVW test and MR-Egger regression consistently indicated no heterogeneity in the majority of causal relationships, as evidenced by Q statistics (*p* > 0.05) (Supplementary Tables 8, 9). Additionally, none of the intercepts derived from the MR-Egger regression analysis significantly differed from zero, providing no indication of horizontal pleiotropy (all intercept *p* > 0.05) (Supplementary Tables 10, 11). The MR-PRESSO test did not reveal any indications of horizontal pleiotropy in the examined causal relationships (*p* > 0.05) (Supplementary Table 12). Moreover, the Leave-one-out analysis demonstrated that individual SNPs did not significantly influence the signals associated with causality, confirming the robustness of the findings (Supplementary Tables 13, 14). Furthermore, in the reverse MR analysis, no supportive evidence was found for a causal impact of IS on gut microbiota/immune cells, providing additional confidence in the established causal relationships (Supplementary Tables 15, 16).

## 4 Discussion

**FIGURE 4:**
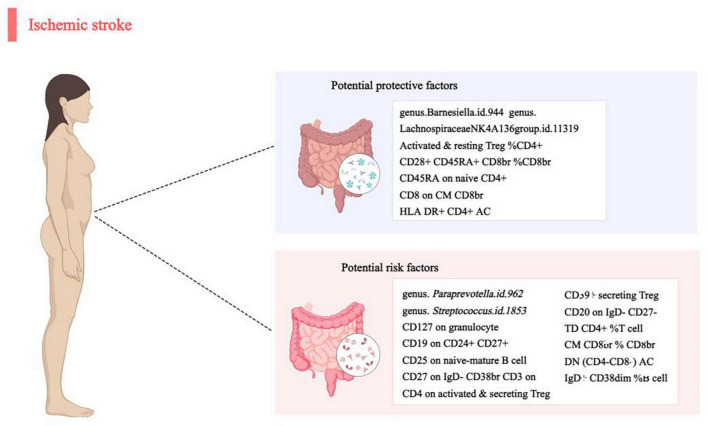
Causal links between gut microbiota, immune cells and ischemic stroke.

In this MR analysis report, we present a pioneering study that establishes a potential causal link between gut microbiota/immune cells and IS. Through a meticulous two-sample MR investigation, we have uncovered compelling evidence of a causal correlation between four specific gut microbial taxa and 16 immune cells concerning IS (Figure 4). This groundbreaking discovery contributes valuable insights into the intricate relationship between gut microbiota, immune cells, and the development of IS. The robustness of our findings, supported by thorough analyses and sensitivity assessments, positions this study as a significant step forward in understanding the potential mechanisms underlying IS.

In our study, we observed a positive correlation between genus.*Paraprevotella.id.962* and genus.*Streptococcus.id.1853* and the risk of ischemic stroke (IS), while genus.*Barnesiella.id.944* and genus.*LachnospiraceaeNK4A136group.id.11319* demonstrated a negative correlation. These findings underscore the potential role of gut microbiota in the development of IS. Dysbiosis in gut microbiota may impact IS through various pathways. Firstly, gut microbiota plays a crucial role in regulating the host immune system, influencing the activity and quantity of immune cells and potentially impacting the inflammatory processes underlying IS. Secondly, certain bacteria may produce metabolites affecting host metabolism, subsequently influencing vascular health and increasing the risk of IS. Additionally, gut microbiota may exert remote effects on the central nervous system by influencing neurotransmitter synthesis and release. In murine models of middle cerebral artery occlusion (MCAO), bacterial imbalance peaked 12–24 h after the ischemic event and gradually returned to normal levels by day 7 (Xu et al., 2021). Studies have shown that stroke-induced changes in mucosal microbiota composition lead to an increase in *Clostridia* and *Akkermansia muciniphila* (Stanley et al., 2018). However, clinical studies have produced varying results regarding taxonomic classification and function. One study found a decrease in *Lactobacillus*, a major type of host probiotic bacteria, following experimental stroke in monkeys (Chen et al., 2019). Recent cohort studies revealed notable enrichment of *Enterobacteriaceae*, *Ruminococcaceae*, and *Akkermansia* at the family level, while *Bacteroidaceae*, *Prevotella*, and *Faecalibacterium* were significantly reduced after IS (Yamashiro et al., 2017). Opportunistic pathogenic Proteobacteria, such as *Enterobacter*, *Megasphaera*, *Oscillibacter*, and *Desulfovibrio*, are more abundant in IS patients with transient ischemic attack (TIA). *E. coli* overgrowth may exacerbate brain infarction via lipopolysaccharide (LPS)-mediated systemic inflammation. Short-chain fatty acids (SCFAs) help maintain gut integrity by reducing inflammation (Tan et al., 2021); depletion of SCFA-producing bacteria like *Roseburia*, *Bacteroides*, *Lachnospiraceae*, *Faecalibacterium*, *Blautia*, and *Anaerostipes* can represent dysbiosis among IS patients due to low fecal SCFA levels (Koh et al., 2016). However, a different study reported that SCFA producers like *Odoribacter* and *Akkermansia* were enriched among IS patients (Li et al., 2019). These diverse findings emphasize the need for further research on the relationship between post-stroke dysbiosis and clinical outcomes.

The most effective method for modulating the gut microbiota is through fecal microbiota transplantation (FMT), a process that involves introducing a suspension of fecal matter from a donor into the recipient’s intestinal tract. FMT has been proven to be highly successful in treating recurrent Clostridioides difficile infections (Duquenoy et al., 2020). Researchers utilizing FMT observed that germ-free mice receiving post-middle cerebral artery occlusion (MCAO) donors’ microbiota had significantly larger brain infarct volumes and reduced behavioral performance compared to those receiving sham-surgery microbiota (Singh et al., 2016). A higher stroke dysbiosis index was associated with more severe brain damage and poorer outcomes (Xia et al., 2019). However, there is still limited understanding regarding the changes in microbiome composition among different enterotypes and their potential impact on the host. All human fecal samples were collected from inpatients after experiencing a stroke, leaving uncertainty about whether these alterations in the gut microbiome occurred before or were induced by stress shortly after the stroke event. Existing clinical studies are primarily cross-sectional, necessitating further longitudinal investigations to uncover the temporal dynamics and remodeling of the microbiome, with the goal of establishing an association between changes in gut microbiota composition and IS. Nonetheless, human microbiota research faces limitations due to significant heterogeneity among subjects. Factors such as genetic variations, dietary habits, lifestyles, age, and co-morbidities/co-medications can all influence the composition of the intestinal flora (Wen and Wong, 2017). These variables highlight the complexity of studying the gut microbiota and emphasize the need for comprehensive and longitudinal investigations to unravel the intricate relationship between microbiome changes and IS.

Immune cells also play a crucial role in the pathogenesis of ischemic stroke (IS). Our study identified 11 types of immune cells positively correlated with the risk of developing IS, while 5 types were negatively correlated. This suggests that the activity and balance of the immune system may have complex effects on the occurrence and progression of IS. Abnormal activation of immune cells can potentially contribute to IS through multiple pathways. Firstly, overactivated immune cells trigger local immune inflammation, leading to endothelial cell damage and ultimately facilitating the development of IS (Fernandez et al., 2019). Secondly, the activity of immune cells affects the coagulation system, increasing the risk of thrombus formation. Additionally, negatively correlated immune cells may reduce the risk of developing IS by regulating immunotolerance and decreasing abnormal immune responses (Li et al., 2022).

B cells have a complex role in the immune response to stroke, exhibiting both harmful and beneficial effects. Following an ischemic stroke, the compromised blood-brain barrier may allow B cells and other peripheral immune cells to enter the affected brain tissue (Malone et al., 2023). These cells play a critical role in regulating the progression of brain injury caused by ischemia and influencing the subsequent inflammatory response after a stroke (Schuhmann et al., 2017; Maheshwari et al., 2023). However, our findings align with this complexity, demonstrating that certain phenotypes associated with B cell activity, such as CD19 on CD24+ CD27+, CD20 on IgD- CD27-, CD25 on naive-mature B cell, CD27 on IgD- CD38br, IgD+CD38dim %B cell are linked to an increased risk of stroke. This highlights the necessity for further investigation into understanding the nuanced role of these specific B cell-related characteristics in strokes.

Similarly, T cells are primarily recognized for their pro-inflammatory effects in ischemic stroke as they infiltrate the blood-brain barrier, choroid plexus, and meninges (Huang et al., 2021). They contribute to the exacerbation of post-stroke inflammation by producing inflammatory cytokines like interferon (IFN)-gamma, interleukin (IL)-21, tumor necrosis factor (TNF), and IL-17 (Meng et al., 2019). The interaction between T lymphocytes and platelets can also worsen microvascular dysfunction and inflammation during ischemic stroke progression (Stoll and Nieswandt, 2019). However, our analysis indicates that T cells have a dual role in stroke pathogenesis due to their regulatory function in inflammation. This regulation is crucial since it impacts both brain injury and repair processes. T cells play a pivotal role in maintaining this balance. Our findings further support the inverse association between CD45RA on naive CD4+ cells and CD8 on CM CD8b with stroke occurrence. Notably, we highlight the significance of CD39 on activated CD4 Tregs as an essential immune element for neuroprotection during ischemic stroke. TD CD4+ %T cell and CM CD8br %CD8br are linked to an increased risk of stroke.

Previous studies suggest that initially, Tregs mitigate acute brain injury by modulating the immune response through their immunosuppressive capabilities and interactions with other immune cells such as neutrophils (Wang et al., 2015). Our findings demonstrate the intricate nature of Treg phenotypes in relation to stroke. While certain Treg phenotypes, such as CD28+ CD45RA+ CD8br %CD8br and Activated & resting Treg %CD4+, exhibit neuroprotective effects, others like CD127 on granulocyte, CD3 on CD39+ secreting Treg, CD4 on activated & secreting Treg, DN (CD4-CD8-) AC are associated with an elevated risk of stroke. This highlights the necessity for further investigation into the nuanced role played by different Treg phenotypes in the context of stroke.

The benefits of this study are notable. MR utilizes genetic variations as substitutes for environmental exposure, establishing a causal link between exposure and disease occurrence. Since genetic variants are assumed to be randomly determined before birth, they are independent of environmental factors, firmly established long before illness onset. This characteristic helps overcome issues associated with residual confounding and reverse causation common in traditional observational studies. Openly accessible datasets were employed in this research, providing more precise estimates and increased statistical power due to extensive sample sizes in GWAS. The findings remained unaffected by horizontal pleiotropy or other variables, ensuring the study’s statistical power to detect a significant association between gut microbiota/immune cells and IS. However, it’s important to acknowledge certain limitations. Firstly, to mitigate population stratification bias, participants of European descent were primarily included, potentially introducing bias into the findings. Secondly, the absence of demographic information such as gender and ethnicity in the original dataset prevented subgroup analyses. Thirdly, due to the insufficient number of SNPs meeting the genome-wide significance threshold (*p* < 5 × 10^–8^), the study focused solely on SNPs reaching the locus-wide significance level (*p* < 5 × 10^–5^). These constraints might limit the generalizability of results and potentially influence the study’s accuracy.

## 5 Conclusion

In summary, our extensive investigations reveal a correlation between specific gut microbiota features and immune cells with the occurrence of IS. Four bacterial characteristics and eleven immune cells exhibit a strong positive relationship with IS, suggesting their potential role as significant contributors. Conversely, two other bacterial features and five immune cells demonstrate an inverse relationship with IS, indicating a potential protective effect. These specific microbial strains and immune cell phenotypes emerge as promising biomarkers, offering valuable insights for the development of innovative strategies in the prevention and treatment of IS.

## Data availability statement

The original contributions presented in this study are included in this article/Supplementary material, further inquiries can be directed to the corresponding authors.

## Author contributions

HS: Writing – original draft, Writing – review & editing. ZW: Writing – original draft. YX: Resources, Writing – original draft. YG: Validation, Writing – original draft. HM: Supervision, Writing – original draft. JG: Funding acquisition, Writing – review & editing.
