# Supplementary material for: Genetically supported causality between gut microbiota, immune cells, and ischemic stroke: a two-sample Mendelian randomization study
Source: Front Microbiol. 2024 Jun 4;15:1402718. doi: 10.3389/fmicb.2024.1402718 (PMC11185428; doi:10.3389/fmicb.2024.1402718)
Supplement: Supplementary file 2 [file Table_2.DOCX]

**STROBE-MR checklist of recommended items to address in reports of Mendelian randomization studies**^1^ ^2^

| **Item No.** | **Section** | **Checklist item** | **Page No.** | **Relevant text from manuscript** |
| --- | --- | --- | --- | --- |
| 1 | **TITLE and ABSTRACT** | Indicate Mendelian randomization (MR) as the study’s design in the title and/or the abstract if that is a main purpose of the study | 1 | Genetically Supported Causality Between Gut Microbiota, Immune Cells, and Ischemic Stroke: A Two-Sample Mendelian Randomization Study |
|  | **INTRODUCTION** |  | 1 | To address this gap, our study aims to meticulously investigate the causal association between gut microbiota/immune cells and the likelihood of developing IS, employing a two-sample Mendelian randomization (MR) analysis. |
| 2 | **Background** | Explain the scientific background and rationale for the reported study. What is the exposure? Is a potential causal relationship between exposure and outcome plausible? Justify why MR is a helpful method to address the study question | 2-3 | Previous studies have highlighted a robust correlation between gut microbiota/immune cells and ischemic stroke (IS). However, the precise nature of their causal relationship remains uncertain. |
| 3 | **Objectives** | State specific objectives clearly, including pre-specified causal hypotheses (if any). State that MR is a method that, under specific assumptions, intends to estimate causal effects | 3 | Previous studies have highlighted a robust correlation between gut microbiota/immune cells and ischemic stroke (IS). |
|  | **METHODS** |  | 3 |  |
| 4 | **Study design and data sources** | Present key elements of the study design early in the article. Consider including a table listing sources of data for all phases of the study. For each data source contributing to the analysis, describe the following: | 4 |  |
|  | a) | Setting: Describe the study design and the underlying population, if possible. Describe the setting, locations, and relevant dates, including periods of recruitment, exposure, follow-up, and data collection, when available. | 4-5 | The summary-level data utilized in this research is available for download. The genome-wide association studies (GWAS) conducted for this study received approval from the relevant institutions, adhering to ethical guidelines. |
|  | b) | Participants: Give the eligibility criteria, and the sources and methods of selection of participants. Report the sample size, and whether any power or sample size calculations were carried out prior to the main analysis | 4-5 | The investigation focused on gut microbiota and immune cells as exposures, with ischemic stroke (IS) as the outcome. All data analyzed were sourced from publicly accessible GWAS. |
|  | c) | Describe measurement, quality control and selection of genetic variants | 5 | (A) a strong association with exposures; (B) no correlation with confounding factors; (C) association with outcomes influenced by the exposures (Ference et al., 2021). |
|  | d) | For each exposure, outcome, and other relevant variables, describe methods of assessment and diagnostic criteria for diseases | 5 | Relevant data on gut microbiota were extracted from the MiBioGen consortium's comprehensive GWAS study, involving 18,340 individuals across 24 cohorts. Utilizing 16S rRNA gene sequencing profiles (Kurilshikov et al., 2021), the analysis focused on 211 taxa, including 131 genera, 35 families, 20 orders, 16 classes, and 9 phyla for mapping microbiome quantitative trait loci. Information on immune traits' summary statistics was publicly accessible through the GWAS Catalog, with accession numbers ranging from GCST0001391 to GCST0002121 (Orrù et al., 2020).  GWAS summary statistics for genetic associations related to IS were obtained from the largest GWAS meta-analysis conducted by the Malik R Lab (Malik et al., 2018). This extensive study included 440,328 individuals of European descent, comprising 34,217 cases and 406,111 controls. |
|  | e) | Provide details of ethics committee approval and participant informed consent, if relevant | 6 | The summary-level data utilized in this research is available for download. The genome-wide association studies (GWAS) conducted for this study received approval from the relevant institutions, adhering to ethical guidelines. |
| 5 | **Assumptions** | Explicitly state the three core IV assumptions for the main analysis (relevance, independence and exclusion restriction) as well assumptions for any additional or sensitivity analysis | 6 | Given the limited number of eligible IVs (genome-wide statistical significance threshold, *p* < 5 × 10^−8^), a locus-wide significance threshold (*p* < 5 × 10^−5^) was adopted for a more comprehensive outcome (Georgakis et al., 2021). To address linkage disequilibrium (LD), a clumping method was employed with parameters set at r^2^ = 0.001 and kb = 10,000. Subsequently, the robustness of the chosen SNPs was assessed by computing the F statistics, utilizing the formula: *F=R^2^(N-k-1)/[(1-R^2^)k]*. In this equation, R^2^ represents the proportion of variability explained by each SNP, N denotes the size of our GWAS sample, and k indicates the number of SNPs. A value of 10 for the F statistic suggests a lack of substantial evidence for instrument bias |
| 6 | **Statistical methods: main analysis** | Describe statistical methods and statistics used | 6 |  |
|  | a) | Describe how quantitative variables were handled in the analyses (i.e., scale, units, model) | 6 | To ensure the reliability and precision of outcomes, a thorough quality check was conducted on SNPs to derive compliant IVs. The selection criteria for SNPs were as follows: (A) a strong association with exposures; (B) no correlation with confounding factors; (C) association with outcomes influenced by the exposures (Ference et al., 2021). |
|  | b) | Describe how genetic variants were handled in the analyses and, if applicable, how their weights were selected | 6 | Given the limited number of eligible IVs (genome-wide statistical significance threshold, *p* < 5 × 10^−8^), a locus-wide significance threshold (*p* < 5 × 10^−5^) was adopted for a more comprehensive outcome (Georgakis et al., 2021). |
|  | c) | Describe the MR estimator (e.g. two-stage least squares, Wald ratio) and related statistics. Detail the included covariates and, in case of two-sample MR, whether the same covariate set was used for adjustment in the two samples | 6 | To address linkage disequilibrium (LD), a clumping method was employed with parameters set at r^2^ = 0.001 and kb = 10,000. Subsequently, the robustness of the chosen SNPs was assessed by computing the F statistics, utilizing the formula: *F=R^2^(N-k-1)/[(1-R^2^)k]*. |
|  | d) | Explain how missing data were addressed | 7 | Find an alternative rs |
|  | e) | If applicable, indicate how multiple testing was addressed | 7 |  |
| 7 | **Assessment of assumptions** | Describe any methods or prior knowledge used to assess the assumptions or justify their validity | 7 | The primary MR analysis utilized the inverse variance-weighted (IVW) method. To assess the reliability of significant findings, sensitivity analyses were conducted using MR-Egger, weighted median, weighted mode, and simple mode |
| 8 | **Sensitivity analyses and additional analyses** | Describe any sensitivity analyses or additional analyses performed (e.g. comparison of effect estimates from different approaches, independent replication, bias analytic techniques, validation of instruments, simulations) | 7 | Cochran's Q statistic and corresponding p-values were employed to assess heterogeneity among selected IVs. In cases where the null hypothesis was not supported, random effects IVW was used instead of fixed-effects IVW. To address potential horizontal pleiotropy, the MR-Egger approach was applied, indicating horizontal multiplicity if the intercept term was statistically significant. The MR-PRESSO method from the MR-PRESSO package. |
| 9 | **Software and pre-registration** |  | 7 |  |
|  | a) | Name statistical software and package(s), including version and settings used | 7 | using R version 4.3.1 |
|  | b) | State whether the study protocol and details were pre-registered (as well as when and where) | 7 | Date：2024.2.10, Yangzhou University |
|  | **RESULTS** |  | 7 |  |
| 10 | **Descriptive data** |  | 7 |  |
|  | a) | Report the numbers of individuals at each stage of included studies and reasons for exclusion. Consider use of a flow diagram | 7 | Scatter plots and funnel plots were employed to assess data integrity, with the former indicating that outliers did not substantially impact results. |
|  | b) | Report summary statistics for phenotypic exposure(s), outcome(s), and other relevant variables (e.g. means, SDs, proportions) | 7 | Funnel plots demonstrated no heterogeneity, confirming the robustness of the correlation. |
|  | c) | If the data sources include meta-analyses of previous studies, provide the assessments of heterogeneity across these studies | 7 | A reverse causality analysis was conducted to evaluate potential reverse causal relationships. |
|  | d) | For two-sample MR:  i.  Provide justification of the similarity of the genetic variant-exposure associations between the exposure and outcome samples  ii.  Provide information on the number of individuals who overlap between the exposure and outcome studies | 7 | Supplementary Tables 2 and 3 |
| 11 | **Main results** |  |  |  |
|  | a) | Report the associations between genetic variant and exposure, and between genetic variant and outcome, preferably on an interpretable scale | 7-8 | Page 7 |
|  | b) | Report MR estimates of the relationship between exposure and outcome, and the measures of uncertainty from the MR analysis, on an interpretable scale, such as odds ratio or relative risk per SD difference | 7-8 | The IVW results indicated that an increased genetic predisposition to the genus. *Paraprevotella.id.962* (OR: 1.106, 95% CI: 1.043-1.172, *p<*0.001), and genus. *Streptococcus.id.1853* (OR: 1.119, 95% CI: 1.034-1.210, *p=*0.005) were associated with an elevated risk of IS. |
|  | c) | If relevant, consider translating estimates of relative risk into absolute risk for a meaningful time period | 7-8 | a lower risk of IS was observed in relation to the genetically predicted abundance of genus.*Barnesiella.id.944* (OR: 0.907, 95% CI: 0.836-0.983, *p*=0.018), and genus.*LachnospiraceaeNK4A136group.id.11319* (OR: 0.918, 95% CI: 0.853-0.983, *p*=0.988) |
|  | d) | Consider plots to visualize results (e.g. forest plot, scatterplot of associations between genetic variants and outcome versus between genetic variants and exposure) | 7-8 | Supplementary Tables 6 |
| 12 | **Assessment of assumptions** |  | 9 |  |
|  | a) | Report the assessment of the validity of the assumptions | 9 | The results of both the IVW test and MR-Egger regression consistently indicated no heterogeneity in the majority of causal relationships, as evidenced by Q statistics (*p* > 0.05) (Supplementary Tables 8 and 9). |
|  | b) | Report any additional statistics (e.g., assessments of heterogeneity across genetic variants, such as *I^2^*, Q statistic or E-value) | 9 | Additionally, none of the intercepts derived from the MR-Egger regression analysis significantly differed from zero, providing no indication of horizontal pleiotropy (all intercept *p* > 0.05) (Supplementary Tables 10 and 11). |
| 13 | **Sensitivity analyses and additional analyses** |  | 9 |  |
|  | a) | Report any sensitivity analyses to assess the robustness of the main results to violations of the assumptions | 9 | The MR-PRESSO test did not reveal any indications of horizontal pleiotropy in the examined causal relationships (*p* > 0.05) (Supplementary Table 12). |
|  | b) | Report results from other sensitivity analyses or additional analyses | 9 | Leave-one-out analysis demonstrated that individual SNPs did not significantly influence the signals associated with causality, confirming the robustness of the findings (Supplementary Tables 13 and 14) |
|  | c) | Report any assessment of direction of causal relationship (e.g., bidirectional MR) | 9 | Reverse MR analysis, no supportive evidence was found for a causal impact of IS on gut microbiota/immune cells, providing additional confidence in the established causal relationships (Supplementary Tables 15 and 16) |
|  | d) | When relevant, report and compare with estimates from non-MR analyses | 9 | None of the intercepts derived from the MR-Egger regression analysis significantly differed from zero, providing no indication of horizontal pleiotropy (all intercept *p* > 0.05) (Supplementary Tables 10 and 11). |
|  | e) | Consider additional plots to visualize results (e.g., leave-one-out analyses) | 9 | Supplementary Tables 10 and 11 |
|  | **DISCUSSION** |  |  |  |
| 14 | **Key results** | Summarize key results with reference to study objectives | 10 | n this MR analysis report, we present a pioneering study that establishes a potential causal link between gut microbiota/immune cells and IS. Through a meticulous two-sample MR investigation, we have uncovered compelling evidence of a causal correlation between four specific gut microbial taxa and 16 immune cells concerning IS |
| 15 | **Limitations** | Discuss limitations of the study, taking into account the validity of the IV assumptions, other sources of potential bias, and imprecision. Discuss both direction and magnitude of any potential bias and any efforts to address them | 14 | However, it's important to acknowledge certain limitations. Firstly, to mitigate population stratification bias, participants of European descent were primarily included, potentially introducing bias into the findings. Secondly, the absence of demographic information such as gender and ethnicity in the original dataset prevented subgroup analyses. Thirdly, due to the insufficient number of SNPs meeting the genome-wide significance threshold (*p* < 5 × 10^-8^), the study focused solely on SNPs reaching the locus-wide significance level (*p* < 5 × 10^-5^). These constraints might limit the generalizability of results and potentially influence the study's accuracy. |
| 16 | **Interpretation** |  | 11 |  |
|  | a) | Meaning: Give a cautious overall interpretation of results in the context of their limitations and in comparison with other studies | 11 | In our study, we observed a positive correlation between genus.*Paraprevotella.id.962* and genus.*Streptococcus.id.1853* and the risk of ischemic stroke (IS), while genus.*Barnesiella.id.944* and genus.*LachnospiraceaeNK4A136group.id.11319* demonstrated a negative correlation. These findings underscore the potential role of gut microbiota in the development of IS. |
|  | b) | Mechanism: Discuss underlying biological mechanisms that could drive a potential causal relationship between the investigated exposure and the outcome, and whether the gene-environment equivalence assumption is reasonable. Use causal language carefully, clarifying that IV estimates may provide causal effects only under certain assumptions | 11 | Immune cells also play a crucial role in the pathogenesis of ischemic stroke (IS). Our study identified 11 types of immune cells positively correlated with the risk of developing IS, while 5 types were negatively correlated. This suggests that the activity and balance of the immune system may have complex effects on the occurrence and progression of IS. |
|  | c) | Clinical relevance: Discuss whether the results have clinical or public policy relevance, and to what extent they inform effect sizes of possible interventions | 12 | B cells have a complex role in the immune response to stroke, exhibiting both harmful and beneficial effects. Following an ischemic stroke, the compromised blood-brain barrier may allow B cells and other peripheral immune cells to enter the affected brain tissue |
| 17 | **Generalizability** | Discuss the generalizability of the study results (a) to other populations, (b) across other exposure periods/timings, and (c) across other levels of exposure | 13 | MR utilizes genetic variations as substitutes for environmental exposure, establishing a causal link between exposure and disease occurrence. Since genetic variants are assumed to be randomly determined before birth, they are independent of environmental factors, firmly established long before illness onset. This characteristic helps overcome issues associated with residual confounding and reverse causation common in traditional observational studies. Openly accessible datasets were employed in this research, providing more precise estimates and increased statistical power due to extensive sample sizes in GWAS. The findings remained unaffected by horizontal pleiotropy or other variables, ensuring the study's statistical power to detect a significant association between gut microbiota/immune cells and IS. |
|  | **OTHER INFORMATION** |  | 15 |  |
| 18 | **Funding** | Describe sources of funding and the role of funders in the present study and, if applicable, sources of funding for the databases and original study or studies on which the present study is based | 15 | This work was funded by the National Natural Science Foundation of China (No. 82172190) . |
| 19 | **Data and data sharing** | Provide the data used to perform all analyses or report where and how the data can be accessed, and reference these sources in the article. Provide the statistical code needed to reproduce the results in the article, or report whether the code is publicly accessible and if so, where | 15 | The datasets presented in this study can be  found in online repositories. The names of the repository/repositories and accession number(s) can be found in the article/Supplementary material. |
| 20 | **Conflicts of Interest** | All authors should declare all potential conflicts of interest | 15 | The authors declare that the research was conducted in the absence of any commercial or financial relationships that could be construed as a potential conflict of interest. |

This checklist is copyrighted by the Equator Network under the Creative Commons Attribution 3.0 Unported (CC BY 3.0) license.

1. Skrivankova VW, Richmond RC, Woolf BAR, Yarmolinsky J, Davies NM, Swanson SA, et al. Strengthening the Reporting of Observational Studies in Epidemiology using Mendelian Randomization (STROBE-MR) Statement. JAMA. 2021;under review.

2. Skrivankova VW, Richmond RC, Woolf BAR, Davies NM, Swanson SA, VanderWeele TJ, et al. Strengthening the Reporting of Observational Studies in Epidemiology using Mendelian Randomisation (STROBE-MR): Explanation and Elaboration. BMJ. 2021;375:n2233.
